# Tuning the Photoresponse of Nano‐Heterojunction: Pressure‐Induced Inverse Photoconductance in Functionalized WO_3_ Nanocuboids

**DOI:** 10.1002/advs.201901132

**Published:** 2019-08-08

**Authors:** Saqib Rahman, Sudeshna Samanta, Alexei Kuzmin, Daniel Errandonea, Hajra Saqib, Dale L. Brewe, Jaeyong Kim, Junling Lu, Lin Wang

**Affiliations:** ^1^ Center for High Pressure Science and Technology Advanced Research Shanghai 201203 China; ^2^ Department of Chemical Physics University of Science and Technology of China Hefei 230026 China; ^3^ HYU‐HPSTAR‐CIS High Pressure Research Center Department of Physics Hanyang University Seoul 04763 Republic of Korea; ^4^ Institute of Solid State Physics University of Latvia Kengaraga street 8 LV‐1063 Riga Latvia; ^5^ Departamento de Física Aplicada‐ICMUV MALTA Consolider Team Universidad de Valencia Edificio de Investigación C/Dr. Moliner 50 Burjassot 46100 Valencia Spain; ^6^ Shanghai Institute of Technical Physics Chinese Academy of Science Shanghai 201800 China; ^7^ X‐Ray Science Division Advanced Photon Source Argonne National Laboratory 9700 South Cass Avenue Argonne IL 60439 USA

**Keywords:** charge carriers, compression, decompression, inverse photoconductivity, nano‐heterojunctions, phase transition, polarons

## Abstract

Inverse photoconductivity (IPC) is a unique photoresponse behavior that exists in few photoconductors in which electrical conductivity decreases with irradiation, and has great potential applications in the development of photonic devices and nonvolatile memories with low power consumption. However, it is still challenging to design and achieve IPC in most materials of interest. In this study, pressure‐driven photoconductivity is investigated in n‐type WO_3_ nanocuboids functionalized with p‐type CuO nanoparticles under visible illumination and an interesting pressure‐induced IPC accompanying a structural phase transition is found. Native and structural distortion induced oxygen vacancies assist the charge carrier trapping and favor the persistent positive photoconductivity beyond 6.4 GPa. The change in photoconductivity is mainly related to a phase transition and the associated changes in the bandgap, the trapping of charge carriers, the WO_6_ octahedral distortion, and the electron–hole pair recombination process. A unique reversible transition from positive to inverse photoconductivity is observed during compression and decompression. The origin of the IPC is intimately connected to the depletion of the conduction channels by electron trapping and the chromic property of WO_3_. This synergistic rationale may afford a simple and powerful method to improve the optomechanical performance of any hybrid material.

## 1 Introduction

In the applications and physics of semiconductors, two general routes are adopted to create the mobile charge carriers in the valence bands (VB) and/or conduction bands (CB): by either impinging illumination (electron–hole pairs) or by applying a gate‐bias through the field‐effect (majority carriers near gate dielectric/semiconductor interface) to increase the material's conductivity by many folds.^1^, ^2^, ^3^ The energy relaxation pathways of photoexcited carriers drive the basic operational principle of ultraviolet (UV) photodetectors,^4^, ^5^ photovoltaics^3^ or solar cells,^2^, ^6^, ^7^ and field‐effect transistor (FET) devices.^6^ Photoconductivity serves as a complementary probe to the regular electrical resistivity and helps us to understand the complex interplay between the light generated carriers, native defects, band‐structure modification, and structural transition relating to the changes in the local coordination environment under external stimuli. A substantial variation in switching the time (photocurrent generation and annihilation) with illumination turned on or off is observed where a slow response often exhibits positive persistent photoconductivity (PrPPC), which has remarkable applications in radiation detectors^8^ and bistable optical switches.^9^ Unlike PrPPC, inverse photoconductivity (IPC), a decrease in the electrical conductivity by light irradiation has also exhibited its potential applications in the development of photonic devices and nonvolatile memories with low power consumption.^10^ IPC has only been observed in semiconductor nanostructures, such as p‐type carbon nanotubes, Si nanowires,^11^ Bi‐doped p‐type ZnSe nanowires, and n‐type InN thin films.^10^ It is very difficult to design and achieve such a unique property in materials of technological interest.

To optimize the photoconductivity gain and improve photodetector yield, various strategies have been adopted. Nanoscale systems offer numerous novel energy relaxation pathways resulting in potentially more efficient devices. Many efforts have been made to improve the absorption/emission performance and charge separation by using nanostructures with high surface‐to‐volume ratio,^12^ layered,^13^, ^14^ and hybrid structure.^15^, ^16^ Especially, heterojunction architectures using both wide and narrow bandgap semiconductors have more flexibilities in tuning the photoresponse and achieving the desired properties.^17^ For instance, n‐type WO_3_ (bandgap ≈2.7 eV) nanocuboids with a coarse layer of p‐type CuO nanoparticles (bandgap ≈1.2–1.4 eV) heterojunction exhibit enhanced visible‐light harvesting due to more effective separation of photogenerated electron–hole pair compared with those in pure WO_3_. The “edged” nanocuboid morphology also favors the highest photoresponse^18^ by reducing the average diffusion time and thereby, reducing the photogenerated electron–hole pairs recombination.

On the other hand, external hydrostatic pressure (*P*) is an effective thermodynamic variable to alter the physical properties of materials by tuning their lattice parameters, including shortening the bond‐lengths and creating distortions in the nearest neighbor environment, electronic structures, and electron–phonon coupling mechanically. Recently, nanodevices inside a diamond‐anvil cell (DAC) with a few layers of MoS_2_ showed synergy between the light and pressure (optomechanical),^19^ and pressure and field‐effect (mechano‐electrostatic) effects.^20^ Besides, the optomechanical evolution of trigonal selenium^21^ and iodine/bromide perovskites^22^ showed a reduction of photoconductivity with increasing pressure due to phase transformation and amorphization. However, the heterojunctions, the basic functional units of detectors have never been studied using external stimuli such as compression. Here, we report for the first time a novel pressure‐induced positive to negative (inverse) photoconductivity transition in an oxide heterostructure semiconductors of n‐type WO_3_ nanocuboids functionalized with p‐type CuO nanoparticles. The appearance of IPC was found to be correlated to the electronic band structure entangled with the lattice distortions as recently predicted.^23^ Pressure‐induced bandgap modifications, WO_6_ octahedral distortion, and the electron–hole pair generation‐recombination process caused a significant increase of photoconductivity in the WO_3_/CuO oxide heterostructures. Our results show that hydrostatic pressure may provide an applicable tool for heterostructure‐based photovoltaic devices, functioning as a switch, controller, or a potential light absorber in future.

## 2 Results and Discussion

### 2.1 Pressure‐Induced Transient Photoresponse

**Figure 1:**
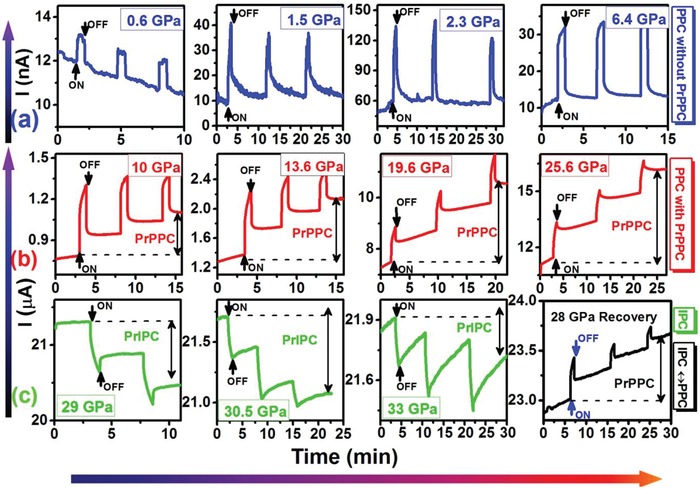
The transient photoresponse with time at low‐bias: a) from 0–6.4 GPa without any PrPPC, b) from 10.6–25.6 GPa with a significant amount of PrIPC, and c) from 29.6–34 GPa with 28 GPa (decompression) with a reversible swing from PPC to IPC.

The WO_3_/CuO heterostructure exhibited photoconductivity, and its transient time‐dependent photoresponses (*I* − *t*) are plotted in **Figure** **1**. The photoactivity of WO_3_ depends on its crystalline structure and preparation methods.^24^ When the CuO nanoparticles are incorporated on the WO_3_ nanocubes, the CuO/WO_3_ heterojunction forms, which leads to a drastic increase in the baseline resistance of the WO_3_ matrix.^25^ High‐pressure photoconductivity measurements were carried out with a direct‐current four‐probe configuration under visible green light illumination with a wavelength of λ = 532 nm (≈2.33 eV) in a diamond anvil cell up to 35 GPa.

We organized the optomechanical behaviors into three different categories (panels in Figure 1a–c) depending on their responses under compression. In the upper panel (a), we noticed only a positive photocurrent (PPC) without any PrPPC. In the middle panel (b), we recorded the PPC with PrPPC and its increment with pressure. Finally, in the lower panel (c), we observed the appearance of IPC along with persistent inverse photocurrent (PrIPC). The right curve in the third panel represents the reversibility of the transition from PPC to IPC followed by decompression. Generally, in nanostructured semiconductors, illumination leads to an increase in the free carrier density resulting from electron–hole pair generation. Since the conduction band level and valence band level of CuO are more negative than the corresponding bands of WO_3,_ 26 electron–hole pairs are created predominantly in CuO, which has a small bandgap (1.2–1.4 eV). Additional details on the creation of electron–hole pairs can be found in the Supporting Information. A heterojunction formed in our system between CuO and WO_3_ results in a large energy barrier consequently, after light irradiation there is weak possibility of electron/hole transfer in any direction (CuO to WO_3_ or reverse), so the electrons and holes recombine after switching off the laser. At 0.6 GPa all charge carriers return to their ground state after switching off the laser. The dark current *I*_dark_ was recorded as 12.3 nA at 0.6 GPa. The *I*_pmax_ was as high as 150 nA at around ≈2.3 GPa without any observable PrPPC where *I*_pmax_ is maximum value of photocurrent when illumination is turned on. Taking into account the Δ*I*/*I*dark, we estimated the increase in photoresponse behavior and observed the maximum value at 2.3 GPa, as shown in Figures 1 and 3a. For details, see the Supporting Information. This change in the pressure‐induced photoresponse is related to structural transitions of WO_3_/CuO, as well as a reduction of the WO_3_ bandgap and its resistivity.^27^ Beyond 2.3 GPa, the characteristic transient response changed significantly, which could be seen in a variation of the rising and decaying time‐constants with pressure. Δ*I*/*I*dark gradually decreases with the further increase of pressure from 6.4 GPa onward, due to the appearance of new phases and WO_6_ octahedra deformation, which creates the mechanisms for trapping the charge carriers.

However, interestingly, the retention of a considerable part of positive photoconductivity occurs above 6.4 GPa, even after the laser was switched off. This PrPPC is clearly shown in Figure 1 and Figure S7b (Supporting Information). At the same pressure, the formation of reduced W^(6−^ *^x^* ^)+^ color centers started to occur. A junction with comparatively smaller work function established, so the electrons and hole can be transferred from one material to the other separately. Above 6.4 GPa, the formation of the reduced layer at the WO_3_ surface decreases the energy barrier between CuO and WO_3_.^27^ At the same pressure, the contact between the CuO and WO_3_ phases is enhanced, allowing for an electron transfer from WO_3_ to CuO. The process is accompanied by the formation reduced W^(6−^ *^x^* ^)+^ color centers and oxygen vacancies at the interface, a phenomenon that usually occurs in oxides under compression.^28^ For details, see the Supporting Information. The vacancies behave as electron traps capturing the electrons generated upon illumination. Consequently, there is less recombination with holes, which leads to the persistent current that is likely produced by holes. Xu et al. demonstrated that after impinging the light, the charge separation makes the electron–hole recombination difficult and initiates the PPC.^29^ Similar phenomena happened in our system beyond 6.5 GPa, where hindrance in electron–hole recombination increased with pressure, and ultimately PPC increased with pressure. Hence, in WO_3_ there will be destruction of majority carriers (electron) and excess of minority carriers (hole). The photocurrent we observed corresponds to the transport of holes, meaning p‐type conduction.

PrPPC increases with the further increase of pressure, as the concentration of trapped charge carriers is also augmented. A considerable amount of PrPPC with longer rising and decaying time‐constants was observed in the middle panel, 10 ≤ *P* ≤ 26 GPa, in contrast to the results depicted in the upper panel. In this region, *I*_dark_ was significantly higher, and the photocurrent increased from a few nA to tens of µA. This change is due to modified band structure of WO_3_/CuO composite and decreased resistivity upon compression. A sudden reduction of pressure‐induced resistivity was also observed in pristine WO_3_ by the increase of carrier concentration due to the appearance of additional energy levels in bandgap.^27^ The sample had a remnant photocurrent as PPC and dark currents were higher than those of the original after repeated on/off laser exposures. However, at the lower pressures (Figure 1a), the photocurrent had good stability with almost full reversibility to its *I*_dark_ values after the light was turned off. We observed maximum PrPPC at 25.6 GPa as shown in Figure 1 and Figure S7c (Supporting Information). After slightly increasing the pressure and switching on the laser, we observed an interesting positive to negative photoconductivity transition. PPC transformed into IPC at 29 GPa, giving rise to significant amounts of PrIPC, which continued up to the highest pressure of 35 GPa, as shown in Figure 1c. Interestingly, such a change from PPC to IPC was reversible with decompression.

The only possibility of IPC is the reverse potential barrier so electrons would prefer to leave CuO. At higher pressure, the formation of a layer of reduced W^(6−^ *^x^* ^)+^ color centers occurs at the interface between the CuO and WO_3_ phases. The bandgap of the reduced surface layer is about 0.7–1.0 eV, so a transfer of electrons will occur from CuO to W^(6−^ *^x^* ^)+^ O_3_ and the current will be caused by the majority carriers (n‐type conduction).^30^, ^31^ This photoresponse phase transformation is in good agreement with the monoclinic transformations, as shown in X‐ray diffraction (XRD) as well as Raman results.

### 2.2 Quantitative Analysis of the Photoresponse

**Figure 2:**
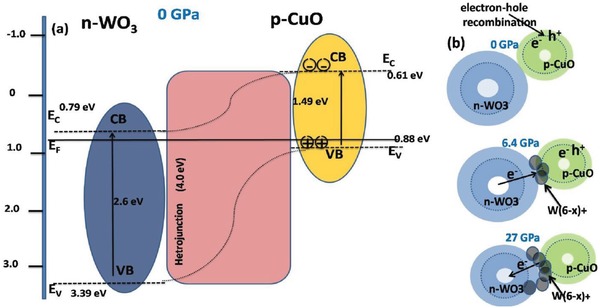
a) Schematic diagram of the photoexcited electron–hole separation process and bandgap representation at ambient conditions. b) Schematic model for the p‐type CuO‐n‐type WO_3_ heterojunction. A formation of reduced W^(6−^ *^x^* ^)+^ color centers at the interface between CuO and WO_3_ phases upon compression.

The response (rising) edge and the recovery (decay) edges consisted of a fast‐response component and a slow‐response component, respectively. The fast‐response component is attributed to the rapid change in carrier concentration as soon as the illumination turned on/off. The slow‐response component corresponds to the defect‐assisted charge carrier trapping/de‐trapping or recombination/generation processes. The time‐dependent responses were fitted (solid lines in Figure S7a, Supporting Information) with a bi‐exponential relaxation equation

(1) $$I\;=I_{0\;}\;+\;I_{1}e^{-t/\tau_{1}}+\;I_{2}e^{-t/\tau_{2}}$$

where *I* is the PC, *I*_0_ is the steady state photocurrent, *t* is the time, *I*_1_and *I*_2_ are constants, τ_1_ and τ_2_ are two relaxation time‐constants as shown with solid lines in Figure S7a (Supporting Information).We marked τ_*r*_ and τ_*d*_ for the rising edge and decaying edges respectively. The rising (decay) edge had two time‐constants of τ_*r*1_ = 0.47 s and τ_*r*2_ = 9.36 s (τ_d1_ = 0.34 s and τ_d2_ = 10.07 s). The three consecutive on/off cycles are shown in **Figure** **2**a inset. In our experiment the calculated incident power radiating on the sample was fixed at about 3.1 mW in order to avoid any thermal effect. A comparison of the ambient relaxation times for different heterojunction systems are provided in Table S1 in the Supporting Information.

**Figure 3:**
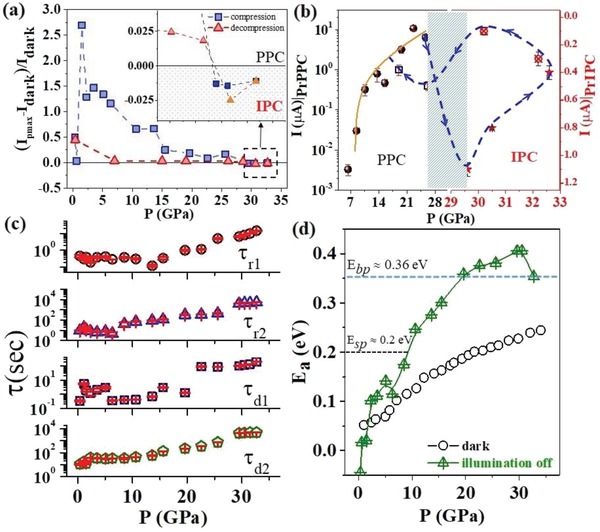
a) The photocurrent gain as a function of pressure, which shows PPC and IPC in the inset. b) Variation of PrPPC and PrIPC and the reversibility of PrIPC as a function of pressure. The shaded region shows the IPC. c) The pressure dependencies of the time‐constants τ for the rising and falling cycles (see text). d) Activation energy *E*_a_ versus pressure plot in the dark and after the illumination was turned off. E_sp_ and E_bp_ correspond to single and bipolaronic activation energies respectively.

The pressure effect on the variation of Δ*I*_ph_ is shown in **Figure** **3**a during compression and decompression. The inset shows the change from PPC to IPC in both pressure cycles. During compression, Δ*I*_ph_ increased to the highest value of 2.7 at 2.3 GPa and then monotonically decreased up to 25 GPa, giving rise to IPC up to 35 GPa. The variation of PrPPC and PrIPC are plotted in Figure 3b, and the solid line shows the fit Δ*I*_ph_ ∝ *P* ^γ^. The reversibility of PPC to IPC and vice‐versa was observed around ≈26–28 GPa. The blue dash line with arrow head points the variation of photocurrent starting from compression at about 26 GPa followed by a reversible transition of photocurrent during decompression around 29 GPa and shaded portion show the pressure range over which we got reversible photocurrent transition (PPC ↔ IPC). The calculated time‐constants for the rising and decaying curves are plotted in Figure 3c. Both τ_r1 _and τ_d1_remained nearly independent with pressure up to 10 GPa, from where a significant increase of both parameters by two orders of magnitude was recorded up to 35 GPa, while both τ_r2_ and τ_d2_ increased by four orders of magnitude. We estimated the thermal activation energy *E*_a_ using the Arrhenius equation at 300 K, and its variation with pressure is plotted in Figure 3d for in the dark and after the illumination was turned off. We observed that the photocurrent and unique PPC ↔ IPC under the combined effect of pressure and illumination in the WO_3_/CuO heterojunction is concomitant to its structural and electronic transitions.

### 2.3 Pressure‐Induced Raman Spectroscopy and XRD

**Figure 4:**
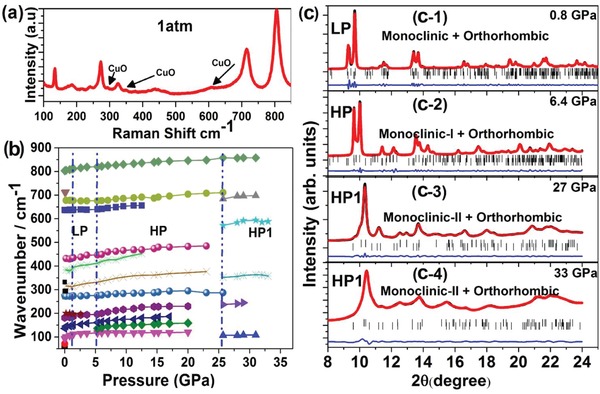
a) Raman spectra of WO_3_/CuO at ambient conditions where the CuO nanoparticles contributions were confirmed. The corresponding modes related to WO_3_ and CuO are marked. b) The variation of the Raman active modes of WO_3_/CuO under compression up to 37 GPa. c) The XRD patterns with Rietveld refinements and different phase coexistence are shown at a few representative pressure points. For simplicity, LP, HP, and HP1 are marked for different pressure regions (see text).

In situ Raman spectroscopy and XRD were carried out to monitor the lattice vibrations and crystal structure of the sample to understand the changes of its photoconductance under high pressure. Raman spectra for the WO_3_/CuO sample are shown in **Figure** **4**a. The spectra were dominated by a WO_3_ monoclinic phase^32^, ^33^ with the presence of a few low‐intensity Raman bands from CuO.^34^ Figure 4b shows the variation of the Raman active modes with pressure up to 40 GPa and matches well with previous reports^33^ confirming several phase transitions (LP, HP, and HP1) around 0.7, 5.5, and 26 GPa. The low‐frequency Raman bands below 200 cm^−1^ are related to the W–O–W bending modes, and the bands in the middle region (200–600 cm^−1^) are attributed to the O–W–O bending modes, whereas the bands in the high wavenumber region above 600 cm^−1^ are related to the W–O stretching vibrations.^33^ The low‐pressure phase transition mainly affects the W–O stretching modes involving oxygen atoms at the apex of the WO_6_ octahedron.

The phase transitions detected by Raman were verified by high‐pressure XRD, as shown in Figure 4c and Figure S1 in the Supporting Information. We found the coexistence of the majority monoclinic (*P*2_1_/*n*) and minority orthorhombic (*Pbcn*) phases at 0.8 GPa.^35^ A thorough analysis of the XRD patterns at all pressures excludes the formation of CuWO_4_, supporting that changes induced in the sample are related to structural changes of WO_3_.^36^ At 6.4 GPa, the LP monoclinic phase (*P*2_1_/*n)* transformed into a HP monoclinic‐I (M1)(*P*2/*c*) phase of WO_3_ and coexisted with the *Pbcn* phase.^37^ Earlier experimental studies and ab initio calculations suggested that these changes are related to a decrease of the W–O–W angles due to WO_6_ octahedral tilting, leading to volume reduction under pressure.^38^ The affected W–O stretching modes involving oxygen atoms also showed expected behavior. At 28 GPa, M1 phase transformed into a monoclinic‐II phase (M2) (*P*2_1_/*c*) with sevenfold tungsten coordination by oxygen atoms.^38^ Beyond 28 GPa, the M2 phase persisted up to 34 GPa. It is clear that the photoconductance changes occurred at similar pressures of phase transitions, suggesting the close relationship between the photoconductivity and crystal structure.

### 2.4 Pressure‐Induced X‐Ray Absorption Spectroscopy (XAS)

**Figure 5:**
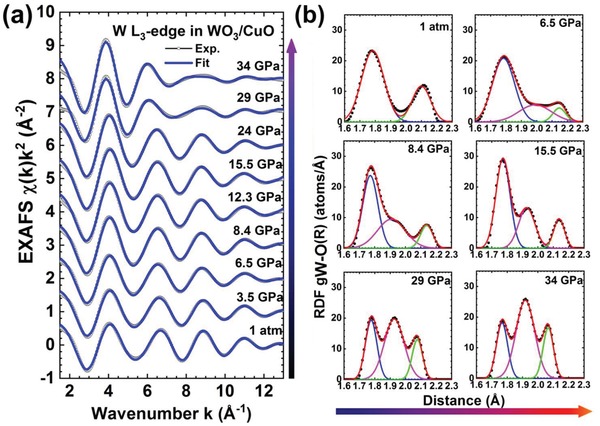
a) The pressure dependence of the experimental and calculated W L_3_‐edge EXAFS for the first coordination shell of tungsten. b) Decomposition of the RDFs *g*_W − O_(R) (solid circles) for the first coordination shell of tungsten into Gaussian components (solid lines). A variation of the WO_6_ octahedra distortion upon compression is well observed.

X‐ray absorption spectra at tungsten (W) L_3_‐edge were collected up to 34 GPa (Supporting Information to gain a deeper understanding on the variation of the local atomic and electronic structures and their relationship with photoconductance. Upon compression, the absorption edge positions remained nearly unchanged suggesting that the effective charge of the tungsten ions is unaltered. However, main resonance (at 10.210 KeV by the transition 2p_3/2_(W) → 5d(W)^39^) intensity increased by 1.7 times along with a change of the fine structures attributed to the structural changes under compression. **Figure** **5**a shows the extended X‐ray absorption fine structure (EXAFS) contributions from the first coordination shell of tungsten, isolated by the Fourier filtering procedure in the *R*‐space range from 0.5 to 2.4 Å, and the true radial distribution functions (RDF) g_W–O_(R) for the W–O atom pairs within the first coordination shell obtained using the regularization method.^40^ RDFs at selected pressure were decomposed into two or three Gaussian contributions and are plotted in Figure 5b. The type of the WO_6_ octahedral distortions and their local environment determined three RDF‐subgroups, as listed in **Table** **1** (G1–G3). At ambient pressure, the WO_6_ octahedra are distorted due to the second‐order Jahn–Teller (JT) effect caused by the W^6+^5d^0^ electronic configuration^41^ that led to the off‐center displacement of the tungsten atoms.

**Table 1:** Types of WO_6_ octahedral distortion at different pressures. Three groups (G1–G3) can be identified

Group	Pressure	Distortion type	Distortion notation
G1 (LP)	1 atm	WO_4_O_2_	4:2
	3.5 GPa	WO_4_O_2_	4:2
	6.5 GPa	WO_4_O_2_	4:2
G2 (HP)	8.4 GPa	WO_3_O_2_O_1_	3:2:1
	12.3 GPa	WO_3_O_2_O_1_	3:2:1
	15.5 GPa	WO_3_O_2_O_1_	3:2:1
	24 GPa	WO_3_O_2_O_1_	3:2:1
G3 (HP1)	29 GPa	WO_2_O_3_O_1_	2:3:1
	34 GPa	WO_2_O_3_O_1_	2:3:1

At ambient pressure, a group of nearest four oxygen atoms was at a distance of 1.8 Å and a group of two distant oxygen atoms was at 2.1 Å (denoted as WO_4_O_2_). Upon increasing pressure to 6.5 GPa, the group of nearest four oxygen atoms remained nearly unchanged, whereas the distribution of the distant oxygen atoms significantly broadened and can be described by two Gaussian peaks with 1.5 and 0.5 oxygen atoms located at 2.0 and 2.15 Å, respectively. This result indicates that the pressure strongly affected the longer W—O bonds, in agreement with the previous studies.^38^

Above 6.5 GPa, the compression resulted in a new peak at about 1.91 Å and led to the narrowing of the other two maxima at about 1.8 and 2.1 Å. The ratio of the peak areas corresponding to the coordination numbers is 3:2:1 (WO_3_O_2_O_1_) in the pressure range of 8.4–24 GPa, and 2:3:1 (WO_2_O_3_O_1_) in the pressure range of 29–34 GPa, encompassing the structural transition in WO_3_. Taking into account that the WO_3_/CuO system is heterogeneous and tungsten atoms can be located in different local environments, the interpretation of the RDF changes for pressures above 6.5 GPa is more complicated. We propose that there are at least two main contributions coming from tungsten atoms. First, those located in compressed WO_3_ give the XRD patterns and are responsible for the peaks in the RDFs at 1.8 and 2.1–2.2 Å. Second, those located in a highly symmetric environment (weakly distorted W^5+^O_6_ octahedron) with an average W—O bond length of about 1.91 Å; such a bond length is typical for reduced tungsten ions and can be found, for example, in cubic Na*_x_*WO_3_.^42^ Within such a model, the samples in the G3 group (Table 1) at pressures above 24 GPa consists of 50% distorted W^6+^O_6_ octahedral units with the distortion type WO_4_O_2_ and 50% regular W^5+^O_6_ octahedra. The distortion of the WO_6_ octahedron and, in particular, a change in the W—O bond lengths affect the position of the CB bottom and hence, changes the bandgap.^38^, ^43^ de Wijs et al. demonstrated that the electronic structure strongly correlates with the distortions of the WO_6_ octahedra and their mutual orientation.^44^ We also observed octahedral distortion in Raman spectroscopy as well as our XAS results, which indicate the structural and electronic transition. The W–O RDFs reconstructed from the EXAFS spectra suggest that in addition to distorted WO_6_ octahedra, regular WO_6_ octahedra appear above 6.5 GPa and their percentage becomes significant (around 50%) above 24 GPa. It has been predicted that the high‐pressure phase could have a bandgap less than 1 eV^23^, ^31^ and would finally close above 27 GPa. Such evidence is corroborated by the n‐type conductivity and the appearance of pressure‐induced chromic properties (light‐gray → dark‐blue) beyond 24 GPa (Figure S5, Supporting Information). The increased amount of structural distortion (Table 1 G3) resulted in the reduction of the tungsten charge state responsible for the blue coloration.^45^, ^46^, ^47^

Photoconductivity measurements along with XAS, XRD, and Raman spectroscopy reveal a significant correlation between the electronic and structural transitions in our metal oxides hybrid structure. Several works have been reported on the origin of negative photoconductivity NPC (IPC) at ambient conditions in doped and undoped semiconductors, functionalized carbon nanotubes and metal nanoparticles by different models. Li et al. elucidated the n‐type enhanced the conducting characteristic (negative photoresponse) in single–walled carbon nanotube‐CdS hybrid nanostructures by photogenerated electron transfer from CdS to carbon nanotubes followed by recombination.^48^ Nakanishi et al. reported that the inverse photoconductance in Au and Ag nanoparticles with molecules comprising the self‐assembled monolayer thin films is due to charge trapping in the transient polaron‐like states.^49^ Gogurla et al. demonstrated the negative photoconductive response of *Bombyx mori* silk protein fibroin hydrogels, triggered by the trapping of charge carrier in Au nanoparticles.^50^ From these observations, it is understood that an inverse photoresponse can be achieved by increasing the recombination or trapping centers for photogenerated charge carriers within the system or by the photogenerated electron transfer across the hybrid nanostructure (heterojunction). In our case, we have unique pressure‐induced inverse photoconductivity that is caused by the increased recombination and decreased trapping of charge carriers as well as structural changes. In the phase transformation from monoclinic‐I to monoclinic‐II during the compression process, the bandgap of WO_3_ is reduced. Above 25 GPa, the electrons prefer to leave CuO due to the decrease of the reverse potential barrier and the reduced bandgap of WO_3_. These electrons will recombine with holes in WO_3_. Hence, due to the increase in the recombination process; there will be obvious inverse photoconductivity beyond 27 GPa. As such, this type of conduction is mainly due to the majority carrier (electron) and can be identified as n‐type conduction. In the decompression process, some of the distortions can be withdrawn, along with the electronic properties, and we recovered PPC from IPC. This result is highly consistent with reversible phase transition confirmed by pressure‐induced Raman and XRD. After releasing pressure, some permanent distortions and defects created by compression impeded the system's recovery to its initial photoresponse. Our results about WO_3_/CuO heterostructure may provide an applicable tool with enhanced performance for photovoltaic devices, functioning as a switch, controller, or a potential light absorber and especially solar water splitting and solar degradation of organic pollutants in future.

## 3 Conclusions

We observed the evolution of pressure‐induced photoconductivity in a heterojunction system of WO_3_/CuO. We revealed the influence of visible photoexcitation, the trapping of charge carriers by oxygen vacancies, pressure‐induced structural transition, and the associated bandgap modification in the heterostructure. Compression gave rise to persistent positive photoconductivity, whereas a remarkable reversible positive‐to‐negative photoconductivity was identified with the synergistic application of pressure and visible light accompanied by the first‐order crystallographic phase transition of WO_3_ with a strong pressure‐induced distortion favored polaronic configurations of tungsten ions. A reduced charge state of tungsten along with the formation of color centers was observed at high pressure. The transition in photoconductivity is mainly related to changes in the structure, bandgap, WO_6_ distortion, and the electron–hole pair recombination process. Although our multidirectional independent studies explain the observed photoconductivity mechanism successfully; a better understanding of controlling the morphology, crystallographic structures, and electrical and optical transport properties of heterojunction nanostructures deserve significant attention for further development.

## 4 Experimental Section

*Sample Synthesis and Scanning Electron Microscopy (SEM)*: The WO_3_/CuO nanocuboids with a molar ratio of Cu:W = 1:20 were successfully prepared by a modified hydrothermal route.^51^ The morphology of 80–150 nm WO_3_/CuO nanocuboids was characterized by scanning electron microscopy and transmission electron microscopy (TEM); see Figure S6a–d (Supporting Information). Figure S6b (Supporting Information) shows the multielemental mapping of the uniform distribution of O, W, Cu, and confirmed the modification of the WO_3_ surfaces with physiadsorbed CuO.

*Photoconductivity and Raman measurements*: In situ four‐probe photoconductivity and resistivity were measured using a Keithley Source‐meter 2410 with a voltage range of 0–0.05 V at room temperature. A DAC was used to generate pressure up to 35 GPa. An insulated gasket was prepared with a mixture of epoxy and cubic boron nitride. Four platinum electrodes were arranged to contact the sample in the chamber, and no pressure medium was used. photoconductivity and Raman measurements were performed using an inVia Renishaw Raman spectrometer system with a laser wavelength of 532 nm with 5× zoom and a grating of 2400 l mm^−1^. A fixed laser exposure time of 20 s was used throughout the photoconductivity experiments.

*X‐Ray Diffraction*: High‐pressure synchrotron X‐ray diffraction experiments were performed at the Shanghai Synchrotron Radiation Facility (SSRF, BL15U1 beamline), China, with an X‐ray wavelength of 0.6199 Å. The ruby fluorescence method was employed for pressure calibration. The 2D XRD images were collected with a MAR 165 detector. The 2D ring type images were integrated using the Dioptas software. The structural analysis was carried out using FULLPROF software.

*X‐Ray Absorption Spectroscopy Measurements*: X‐ray absorption spectra were acquired in the transmission mode on beamline 20BMB at the Advanced Photon Source. A powder sample was placed into a DAC, and silicon oil was used as a pressure medium. Extended X‐ray absorption fine structure and X‐ray absorption near edge structure (XANES) were extracted from the experimental signals using the conventional approach implemented in the EDA package.^52^

## Conflict of Interest

The authors declare no conflict of interest.

## Supporting information

Supplementary
