# Supplementary material for: Tuning the Photoresponse of Nano‐Heterojunction: Pressure‐Induced Inverse Photoconductance in Functionalized WO3 Nanocuboids
Source: Adv Sci (Weinh). 2019 Aug 8;6(19):1901132. doi: 10.1002/advs.201901132 (PMC6774034; doi:10.1002/advs.201901132)
Supplement: Supplementary file 1 — Supplementary [file ADVS-6-1901132-s001.pdf]

Copyright WILEY-VCH Verlag GmbH & Co. KGaA, 69469 Weinheim, Germany, 2019.

ADVANCED SCIENCE
Open Access

Supporting Information

for *Adv. Sci.,* DOI: 10.1002/advs.201901132

Tuning the Photoresponse of Nano-Heterojunction: Pressure-Induced Inverse Photoconductance in Functionalized WO_3 Nanocuboids

*Saqib Rahman, Sudeshna Samanta, Alexei Kuzmin, Daniel Errandonea, Hajra Saqib, Dale L. Brewe, Jaeyong Kim, Junling Lu, and Lin Wang**

Supplementary Information

Tuning the photoresponse of nano-heterojunction: Pressure-induced inverse photoconductance in functionalized WO_3 nanocuboids

S. Rahman[1,2†], Sudeshna Samanta[1,3††], Alexei Kuzmin[4], D. Errandonea[5], Hajra Saqib[6,1], Dale L. Brewe[7], Jaeyong Kim[3], Junling Lu[2], Lin Wang[1*]

[1]*Center for High Pressure Science and Technology Advanced Research, Shanghai 201203, China.*
[2]*Department of Chemical Physics, University of Science and Technology of China, Hefei, China.*
[3]*HYU-HPSTAR-CIS High Pressure Research Center, Dept. of Physics, Hanyang University, Seoul, Korea.*
[4] *Institute of Solid State Physics, University of Latvia, Kengaraga street 8, LV-1063 Riga, Latvia.*
[5]*Departamento de Física Aplicada-ICMUV, MALTA Consolider Team, Universidad de Valencia, Edificio de Investigación, C/Dr. Moliner 50, Burjassot, 46100 Valencia, Spain.*
[6]*Shanghai Institute of Technical Physics, Chinese Academy of Science, Shanghai 201800, China.*
[7]*X-Ray Science Division, Advanced Photon Source, Argonne National Laboratory, 9700 South Cass Avenue, Argonne, Illinois 60439, USA*

More characterizations

S1. Electron-hole pairs generation:

Here, we used a 532 nm laser with a photon energy of (E=hc/λ, 2.34 eV) that is ~~little~~ smaller than the band gap of WO_3 (2.6 eV) but much higher than that of CuO (1.2-1.4 eV), and can easily generate electron-hole pairs, introducing a positive or negative photoelectric response depending on a variation of the band gap and hetero-junction with pressure. As CuO has a band gap smaller than the laser energy, the electron-hole pairs are mainly generated in CuO at ambient conditions. By increasing pressure, the band gap of WO_3 decreases[1] but the material remains as a semiconductor at least up to 25 GPa.[21, 25] On the other hand, by comparing CuO with isomorphic AgO, which has similar compressibility, one can predict that the band gap of CuO would not collapse within the pressure range of our interest[2], and might decrease slowly. Thus, changes in the electronic structure of WO_3 will dominate changes in the photoresponse. In particular, the above described subtle change in W-O-W interatomic bonding and WO_6 octahedral tilting, can favor a pressure-induced band-gap reduction in WO_3 that is consistent with the dramatic increase in the photoresponse (270%) at 2.3 GPa.

S2. PPC and trapping charge carrier:

The high-pressure phase of WO_3 adopted a highly distorted structure where the appearance of a new peak and elongation of the W-O bonds were confirmed by XAS spectra (Table 1: G2). Such an elongation helps to increase the oxygen vacancy concentration by decreasing the vacancy formation energy, which leads to the slow recovery of charge carriers and agrees very well with our observed PrPPC (Figure 1(b) and Figure 3(c)). Xu *et al.* demonstrated that after impinging the light, the charge separation makes the electron-hole recombination difficult and initiates the

PPC[3]. Similar phenomena happened in our system beyond 6.5 GPa, where hindrance in electron-hole recombination increased with pressure, and ultimately PPC increased with pressure.

S3. X-ray diffraction at ambient conditions and after decompression

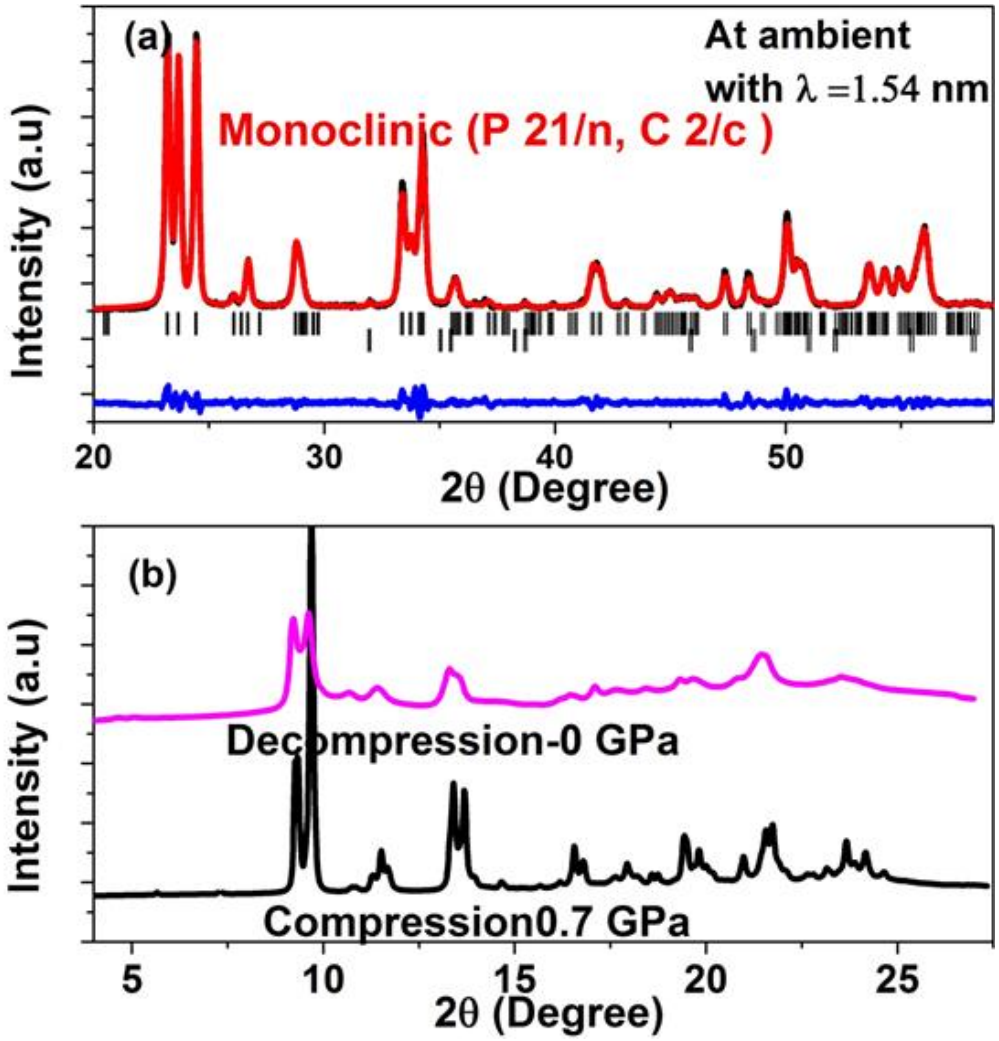

Figure S1. (a) XRD pattern of WO_3/CuO nanocubes at ambient pressure. (b) Comparison of XRD patterns of WO_3/CuO nanocubes measured during compression and after decompression.

S4. Decompression of high-pressure Raman spectroscopy

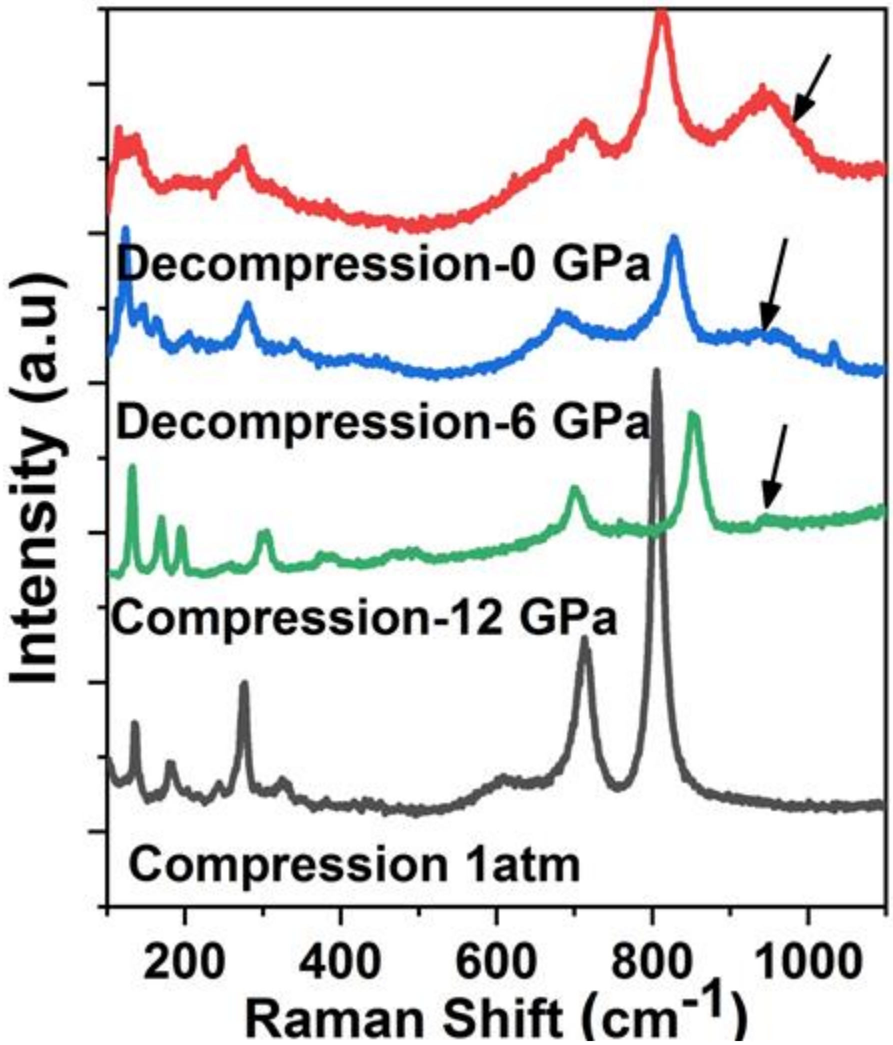

Figure S2. A comparison of the Raman scattering spectra of the compressed and decompressed WO_3/CuO nanocubes with an indicating peak at 950 cm$^{-1.}$ Arrows indicate the positions of the characteristic vibrational bands of W^{5+}-O and/or double W-O bonds.

The band at 950 cm^{-1} is due to W^{5+}-O bonds at 12 GPa under compression and 6 GPa under decompression, however, at 0 GPa after decompression it is mainly due to the W^{6+}-O bonds. The broadening of this band at 0 GPa after decompression correlates well with the Bragg's peak broadening in Fig. S1(b).

S5. X-ray absorption spectroscopy measurements

The pressure dependence of the W L_3-edge XANES spectra is shown in Fig. S3, and the experimental W L_3-edge EXAFS spectra and their Fourier transforms are presented in Fig. S4. The contribution from the first coordination shell of the tungsten atoms (Fig. 5(a) Manuscript) was isolated by the Fourier filtering procedure in the R-space range from 0.5 to 2.4 Å, and the true radial distribution function (RDF) $g_{W-O}(R)$ for the W-O atom pairs (Fig. 5(b) Manuscript) was obtained using the regularization method,[4] which has an advantage in cases of arbitrary structural disorder.[61] The calculations were performed using theoretical scattering amplitude and phase shift functions for the W-O atom pair obtained by the ab initio real-space multiple-scattering code FEFF8.5L.[63] The photoelectron inelastic losses were accounted for within one-plasmon approximation using the complex exchange-correlation Hedin-Lundqvist potential.[64] The best-fit of the experimental W L_3-edge EXAFS signals from the first coordination shell (Fig. 5(a) Manuscript) was performed in the k-space range from 1.5 to 13 $Å^{-1}$. The amplitude of the theoretical EXAFS spectra was scaled by the constant factor S_0^2=0.69.

The obtained RDFs $g_{W-O}(R)$ were decomposed into two or three Gaussian contributions (Fig. 5(b) Manuscript), which were used to estimate the type of WO_6 octahedra distortion and its variation upon compression. The obtained results allowed us to separate all RDFs into three groups G1-G3 (Table 1 manuscript), corresponding to the three types of average local environment around the tungsten atoms.

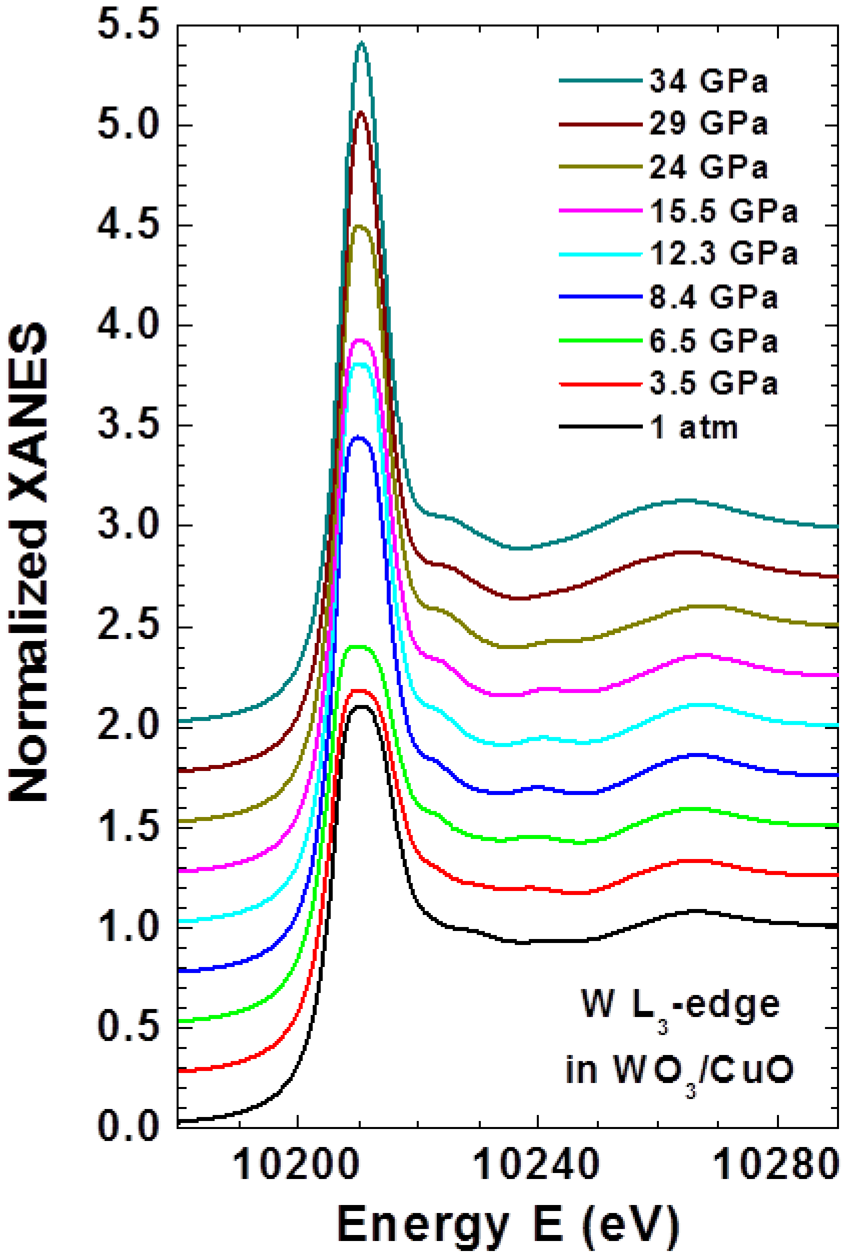

Figure S3. Variation of the normalized x-ray absorption near edge structure (XANES) μ(E) at the W L_3 edge of WO_3/CuO. The spectra are vertically displaced for clarity.

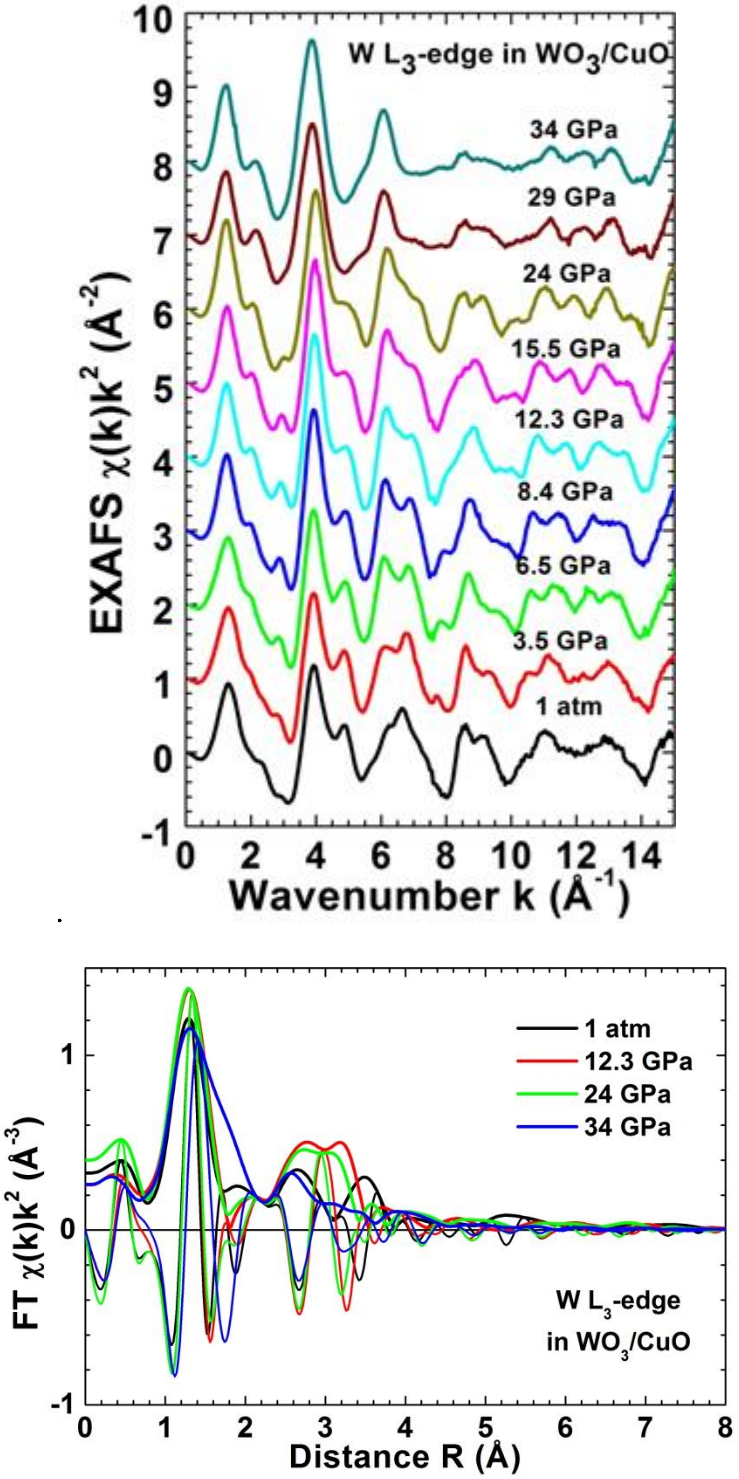

Figure S4. Pressure dependence of the W L_3-edge EXAFS spectra of WO_3/CuO and their Fourier transforms.

S6. Color variation through low-resolution optical microscope images

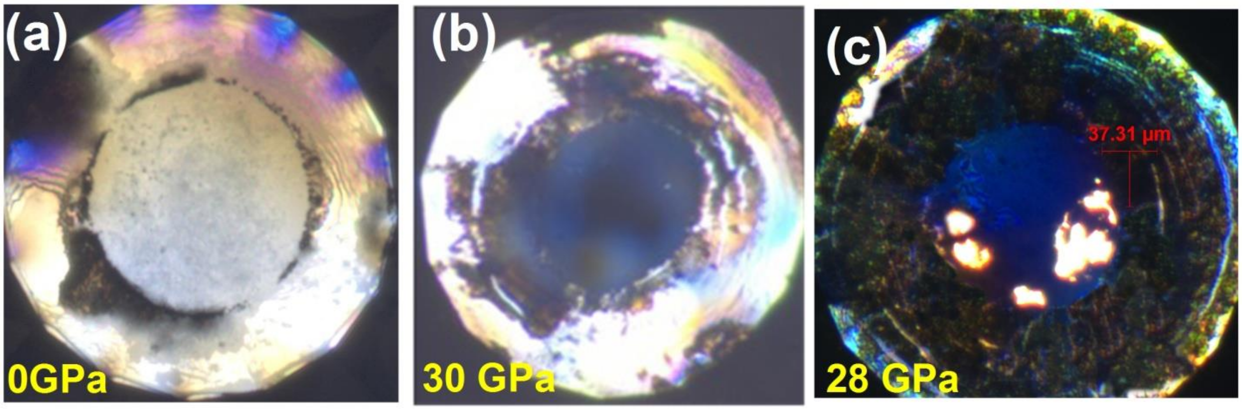

Fig. S5. Color variation through low-resolution optical microscope images captured in WO_3/CuO for 0 and 30 GPa without any pressure media (a-b) and after with pressure media at 28 GPa (c)

It is known that the oxidation state of tungsten in WO_3 is 6+ but at high pressure, it can turn to a bluish color that is due to the formation of W^{5+} color centers by gaining electrons[5,6].

S7. Comparison of different heterojunction systems under different illumination wavelengths at ambient conditions

	**Heterojunction systems under ambient conditions**	**Illumination wavelength (λ nm)**	τ_r **(sec)**	τ_d **(sec)**	ΔI_{ph}
1.	WO_3 nanoparticles[7]	365	30.3	218.0	58
2.	CuO nanoparticle	480	26	28	2.1
3.	WS_2/WO_3[8]	633	0.1	60	9.0
4.	ZnO/CuO[9]	white-light	4.2	5.2	1.1
5.	CuO/Si	532			4.5
6.	**WO_3/CuO (this work)**	**532**	**0.47**	**10.07**	**0.47**

Table S1: Comparison of different heterojunction systems under different illumination wavelengths at ambient conditions.

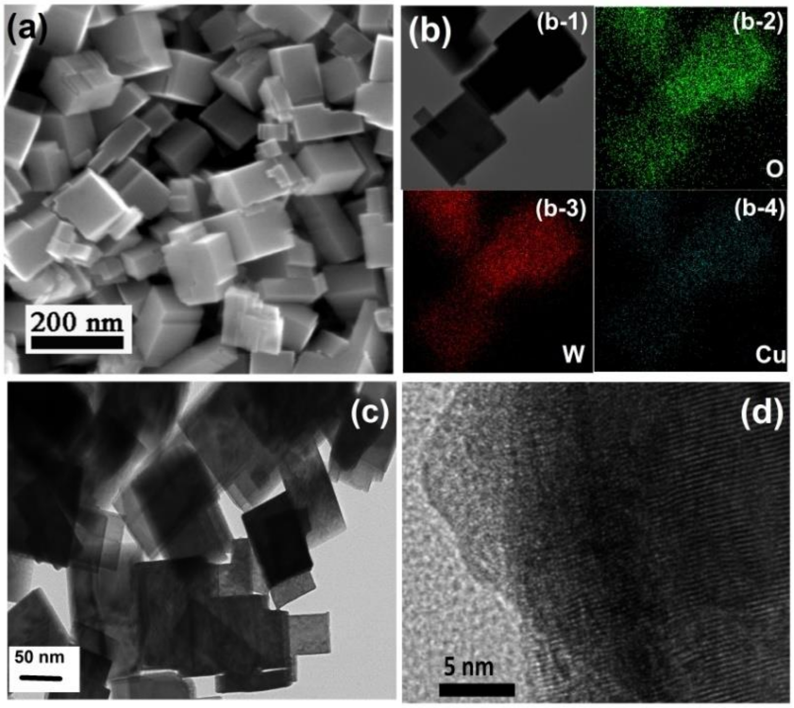

Figure S6 (a) SEM image and (b) multi-elemental mapping images CuO/WO_3 hybrids. (c-d) HTEM images of WO_3 nanocubes decorated with a CuO nanoparticle**.**

S8: Relation between the high pressure and the carrier density of WO3

Upon compression, band structures and band gap changes, so it is obvious the carrier density will change. Because the band gap was reduced, the effective mass is modified. Mostly in high pressure, with change of band gap the carrier density change. In WO3, resistivity decreases with pressure as we have discussed in main text and we have added new result (See Figure S9), so obviously carrier density will be change. The pressure induced resistivity drop is caused by an increase in carrier concentration, which is related to the increase of the additional energy levels in energy band gap.

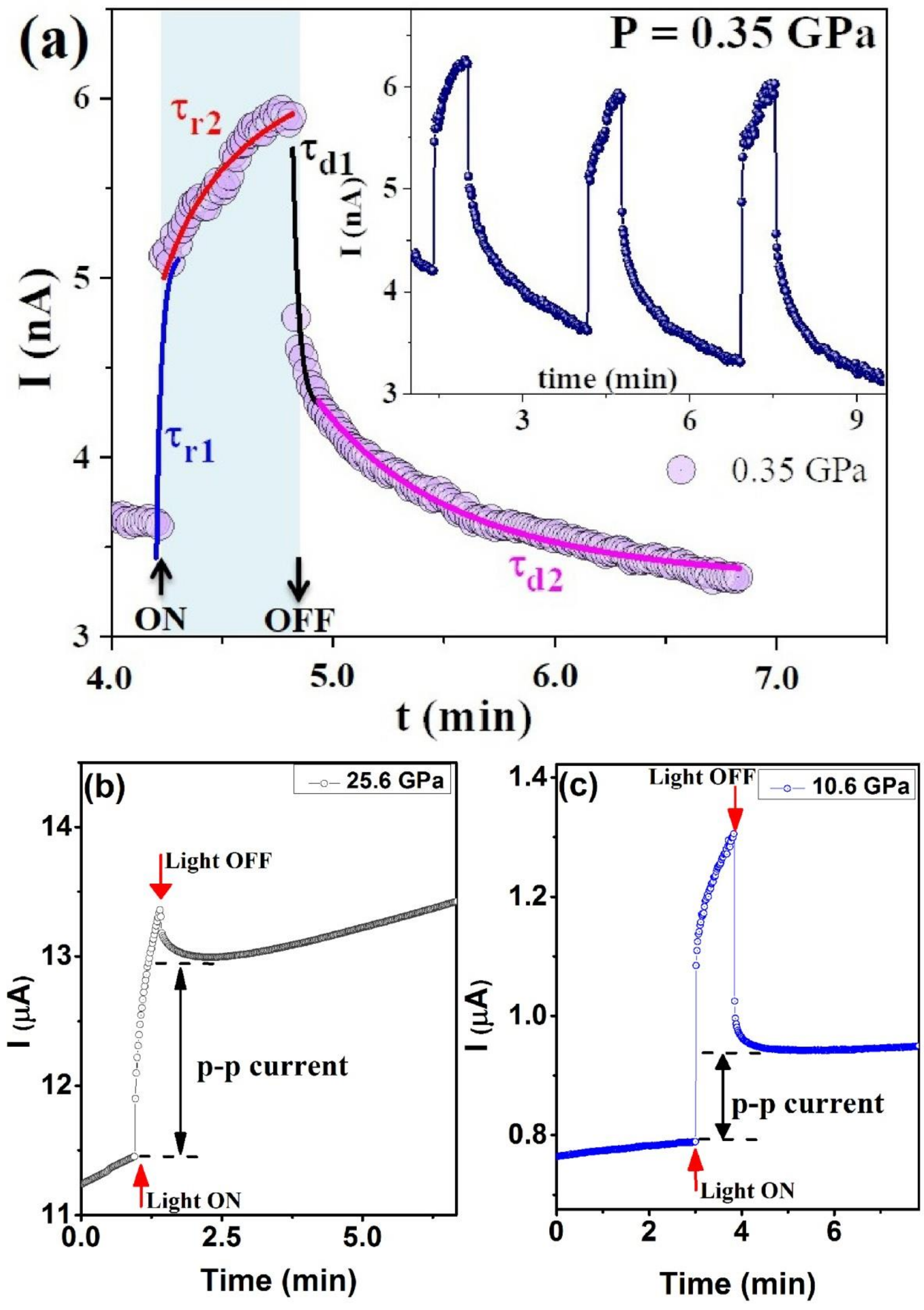

Figure S7: (a) Transient photocurrent-time data at a low pressure $P = 0.35$ GPa where solid lines show the fits using equation 1. The inset shows three consecutive cycles demonstrating the reproducibility of the data. **(b-c)** PPC at 25.6 GPa and 10 GPa.

S9: Schematic diagramed of band gap of WO_3/CuO nanostructure at High pressure

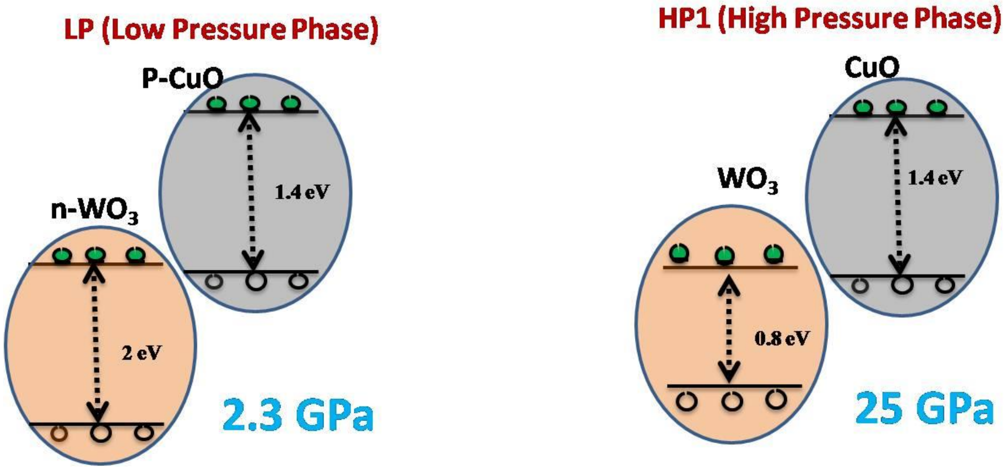

Figure S8: Schematic diagramed of band gap of WO_3/CuO nanostructure at 2.3 GPa and 25 GPa.

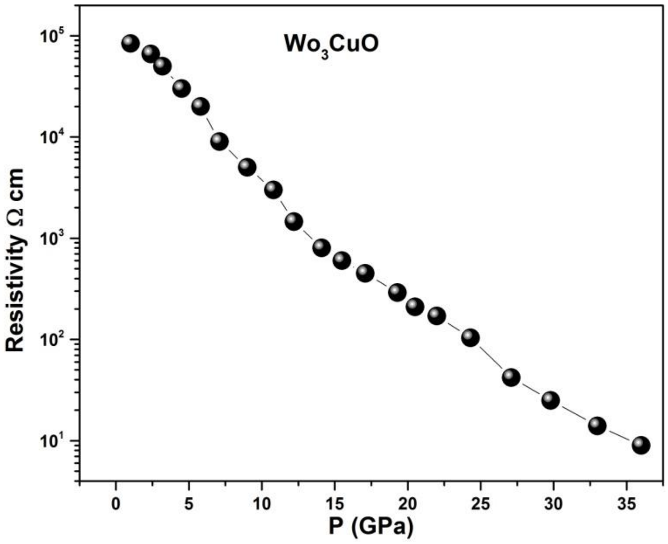

Figure S9: Pressure induced electrical resistivity of WO_3/CuO nanostructure

References:

1 Li, Y. *et al.* High-Pressure Electrical Transport Behavior in WO3. *The Journal of Physical Chemistry C* **116**, 5209-5214, doi:10.1021/jp210559c (2012).

2 Grzelak, A. *et al.* Persistence of Mixed and Non-intermediate Valence in the High-Pressure Structure of Silver(I,III) Oxide, AgO: A Combined Raman, X-ray Diffraction (XRD), and Density Functional Theory (DFT) Study. *Inorg. Chem.* **56**, 5804-5812, doi:10.1021/acs.inorgchem.7b00405 (2017).

3 Xu, J. *et al.* Electric-field effects on persistent photoconductivity in undoped n-type epitaxial GaN. *Appl. Phys. Lett.* **88**, 072106, doi:10.1063/1.2174841 (2006).

4 Kuzmin, A., Anspoks, A., Kalinko, A., Timoshenko, J. & Kalendarev, R. External pressure and composition effects on the atomic and electronic structure of SnWO4. *Sol. Energy Mater. Sol. Cells* **143**, 627-634, doi:10.1016/j.solmat.2014.12.003 (2015).

5 Makwana, N. M. *et al.* A simple and low-cost method for the preparation of self-supported TiO2-WO3 ceramic heterojunction wafers. *Journal of Materials Chemistry A* **2**, 17602-17608, doi:10.1039/c4ta03257h (2014).

6 Leftheriotis, G., Papaefthimiou, S., Yianoulis, P. & Siokou, A. Effect of the tungsten oxidation states in the thermal coloration and bleaching of amorphous WO3 films. *Thin Solid Films* **384**, 298-306, doi:http://doi.org/10.1016/S0040-6090(00)01828-9 (2001).

7 Huo, N., Yang, S., Wei, Z. & Li, J. Synthesis of WO3 nanostructures and their ultraviolet photoresponse properties. *J. Mater. Chem. C* **1**, 3999-4007, doi:10.1039/c3tc30527a (2013).

8 Huo, N., Yue, Q., Yang, J., Yang, S. & Li, J. Abnormal Photocurrent Response and Enhanced Photocatalytic Activity Induced by Charge Transfer between WS2 Nanosheets and WO3 Nanoparticles. *ChemPhysChem* **14**, 4069-4073, doi:doi:10.1002/cphc.201300680 (2013).

9 Wang, P., Zhao, X. & Li, B. ZnO-coated CuO nanowire arrays: fabrications, optoelectronic properties, and photovoltaic applications. *Opt. Express* **19**, 11271-11279, doi:10.1364/oe.19.011271 (2011).
